# Impact of Electronic Health Record Systems on Prescribing Errors in Pediatric Clinics

**DOI:** 10.3390/healthcare7020057

**Published:** 2019-04-05

**Authors:** Brooke L. Gildon, Michelle Condren, Christine C. Hughes

**Affiliations:** 1 College of Pharmacy, Southwestern Oklahoma State University, Weatherford, OK 73096, USA; 2 School of Community Medicine, University of Oklahoma, Tulsa, OK 74135, USA; michelle-condren@ouhsc.edu; 3 Saint Francis Health System, Inc., Tulsa, OK 74136, USA; cchughes@saintfrancis.com

**Keywords:** pediatrics, children, prescribing errors, medication, electronic health records

## Abstract

Medication errors are commonly reported in the pediatric population. While evidence supports the use of e-prescribing to prevent certain errors, prescribing with an electronic health record (EHR) system is not devoid of errors. Furthermore, the majority of EHRs are not equipped with functionalities addressing pediatric needs. This study analyzes three unique EHRs in three pediatric clinics. It describes the functionality of each system and identifies errors found in e-prescribed prescriptions. Finally, the study estimates the proportion of e-prescribing errors that could have been avoided if those EHRs had met requirements set by the American Academy of Pediatrics (AAP). The number of prescriptions reviewed for Clinics 1, 2, and 3, respectively, were: 477, 408, and 633 with total error rates of 13.2%, 8.8%, and 6.6%. The clinic EHRs included 21%, 26%, and 47% of the AAP pediatric requirements for safe and effective e-prescribing for children. If all AAP elements had been included in the EHRs, over 83% of errors in the examined e-prescriptions could have been prevented. This study demonstrates that EHR systems used by many pediatric clinic practices do not meet the standard set forth by the AAP. To ensure our most vulnerable population is better protected, it is imperative that medical technology tools adequately consider pediatric needs during development and that this is reflected in selected EHR systems.

## 1. Introduction

The Joint Commission (Oakbrook Terrace, IL, USA) reports that errors linked to medications are believed to be the most common medical related errors [1]. Furthermore, they account for a significant cause of preventable adverse events [1]. Medication errors also occur more frequently in the pediatric population compared to adults and convey a higher risk of adverse effects in children because there is often a narrower therapeutic window and pediatric patients are often unable to communicate adverse effects [1,2]. Common pediatric medication errors include improper dose or quantity, omission error, and wrong drug; all of which could also be encountered with electronic prescribing (e-prescribing). While there may be evidence to support that e-prescribing mitigates certain medication errors, especially errors associated with handwritten prescriptions, it is clear that e-prescribing also contributes to a new class of medication errors [3,4]. For example, there are often limited or no rules related to drug selection, standardization of weight/use of weight in the electronic health record (EHR), or dose formulation identification. Furthermore, a systematic review of information technology (IT) interventions revealed that only one-half of the randomized controlled trials studied demonstrated that e-prescribing resulted in a significant reduction of medication errors [4].

Since 2008, there has been significant growth in the adoption of EHR and e-prescribing as tools to increase the quality and safety of health care [5]. The US Department of Health and Human Services reported that more than 50% of eligible professionals and 80% of eligible hospitals have adopted these systems [5]. Specific to office-based pediatricians, the use of EHRs increased from 58% in 2009 to 79% in 2012 [6]. However, only 14% used a fully functional EHR and even fewer added optional pediatric functionality [6]. As more health care facilities strive to meet criteria for meaningful use core measures of their EHR as required by the Centers for Medicare and Medicaid Services, the use of e-prescribing is likely to continue to increase [7]. Meaningful use of the EHR is based on five pillars of health outcome policy priorities with “improving quality, safety, efficiency, and reducing health disparities” as one of the pillars that directly impacts medication safety. One measure that demonstrates adoption of this pillar is to generate and transmit permissible prescriptions electronically. However, the unique requirements of pediatric patients are often not adequately addressed by most commonly used adult-focused information systems [2]. Poorly developed systems, as well as suboptimal implementation strategies, are just a few contributors leading to suboptimal use of EHRs for e-prescribing in children [2,8,9]. Additionally, many systems do not adequately educate and support providers and clinics on the customizable options within the EHR as they relate to the pediatric population leading to e-prescribing and medication errors.

While e-prescribing may reduce pediatric medication errors, pediatric practices have historically been slow adopters of e-prescribing with few of those adopters actually using a pediatric-supportive system [10]. More evidence must be gathered about the types and incidences of medication errors in pediatric outpatient settings that relate to specific EHR systems in order to encourage adoption of e-prescribing and improve effectiveness and safety.

In support of the American Academy of Pediatrics (AAP) recommendation that providers working with children adopt e-prescribing systems with pediatric functionality [11], this study (1) describes the functionality and typology of EHR systems in use by three unique clinics that care for children and (2) classifies the type and incidence of prescribing errors observed with each of these unique systems. Lastly, the study (3) estimates the proportion of e-prescribing errors that could have been avoided if those EHRs had met requirements set by the AAP. This study will advance practices currently using e-prescribing toward stage 3 meaningful use criteria as established by US Department of Health and Human Services. Specifically, addressing ways to prevent pediatric e-prescribing errors will lead to improving quality, safety, and efficiency for improved health outcomes.

## 2. Materials and Methods

A cross-sectional, descriptive study design was carried out with three clinics representing three unique EHR systems and practice settings. Including three unique systems provides both depth and breadth as we define systems’ characteristics and capabilities. To be included, clinics must provide care for children, utilize an EHR, and transmit electronic prescriptions to community pharmacies. To estimate a 10% error rate (as observed in prior research [12]) with 5% precision and 95% confidence, a sample size of 139 prescriptions was needed [13]. However, to further improve generalizability and ensure adequate identification of potential errors linked to e-prescribing, the study chose to review at least 400 new prescriptions per clinic. All prescriptions written from each clinic were reviewed for a 1–2 month period, depending on the time needed to meet or exceed 400 new prescriptions. Investigators identified a point person at each clinic who worked with the research team to facilitate access to information and completion of paperwork needed to obtain EHR access; and identify specifics about their clinic’s EHR and e-prescribing functionalities. Data were entered into a Microsoft Excel^®^ 2016 database (Microsoft Corp., Redmond, WA, USA) for analysis. All results were de-identified with no link to protected health information (PHI) and the study was conducted in accordance with the Declaration of Helsinki. The protocol was approved by the University of Oklahoma Health Sciences Center Institutional Review Board (#3463).

Three clinics within the same community were included in the study. Each clinic represented an office within a bigger system with a unique EHR. Clinic 1 is a pediatric clinic within a Federally Qualified Health Center (FQHC) which was the second largest FQHC in the state. Clinic 1 is not a teaching facility and was staffed by three pediatricians. All prescriptions written for a 2-month period were included in the review. Clinics 2 and 3 are both part of university-based health systems representing different state universities, but similar in that they are teaching facilities staffed by trainees/medical residents, nurse practitioners or physician assistants, and faculty physicians. Clinic 2 is a family medicine clinic (only prescriptions for pediatric patients were included in the study) and Clinic 3 represents a pediatric clinic. To obtain the needed pediatric prescriptions to review for Clinic 2, two months of review was required. Whereas, Clinic 3 only required the review of one month of prescription data. EHRs analyzed include Success EHS (Clinic 1), Centricity™ EMR (Clinic 2), and Epic (Clinic 3).

To accomplish study aim 1, each of the three clinic EHR systems was assessed for its compliance with the AAP pediatric requirements for safe and effective e-prescribing in the categories of patient information, medication information, cognitive support, pharmacy information and data transmission [11]. The nineteen elements related to the AAP pediatric requirements are described in Table 1. The presence or absence of each pediatric requirement within each EHR was documented as “met”, “partially met”, or “not met”. In addition, each practice setting was evaluated for overall compliance with the AAP requirements.

For study aim 2, over 400 new prescriptions from each practice setting were used to identify and classify e-prescribing errors by reviewing the name of medication, patient age, patient weight, medication directions/dose, quantity, and indication. The method for prescription review has been previously described [12]. Prescribing errors were identified by type based on a rubric from previously published literature in the area of medication errors (i.e., incomplete or inadequate prescription, dosing outside of the recommended range, drug selection, documentation, and administration method) [12]. Every error represents a unique, single prescription. If a prescription contained more than one error type, it was only counted as one erroneous prescription. Table 2 provides examples of each error type. A dosing error was classified as such if a medication dose deviated by more than 10% from the indicated dosage range based on Pediatric Lexi-Comp or primary literature support [12]. Errors identified were also described based on their medication class (e.g., antihistamines, antimicrobials, respiratory medications). For study aim 2, an expert panel comprised of pediatric-trained pharmacists and physicians was used to confirm medication errors. Errors were independently identified by two members of the expert panel and a third person adjudicated any differences. The prescription error rate was reported as a proportion by each type of EHR/e-prescribing system.

Finally, the resulting medication errors were reviewed to determine if they could have been prevented if the EHR system had met AAP recommendations. As with study aim 2, two expert panel members independently completed the review with a third adjudicating any differences.

## 3. Results

**Figure 1:**
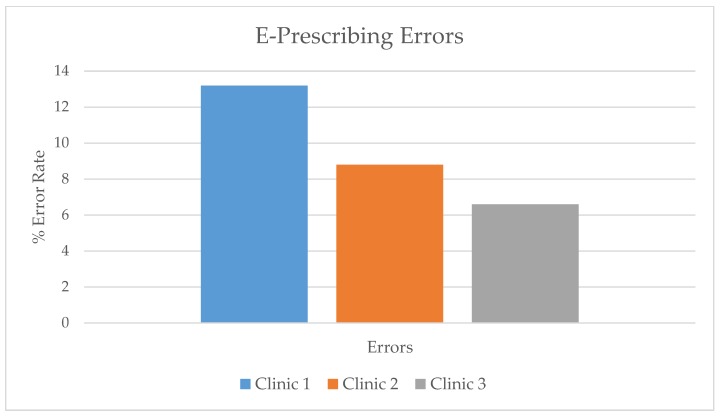
Total e-prescribing errors per clinic.

All three clinics provided a list of de-identified, retrospective prescriptions that represented either one or two months of prescription data to meet or exceed the study-determined 400 prescriptions. The number of prescriptions reviewed for Clinics 1, 2, and 3, respectively were: 477, 408, and 633 with total error rates of 13.2%, 8.8%, and 6.6%, respectively (Figure 1). Errors of various types occurred in almost every drug class. The medication classes with the highest percentage of errors were antimicrobial and respiratory agents. As an example, an overdose error in an amoxicillin prescription was classified as an error in the antimicrobial drug class. Of the errors found, 27%, 24%, and 33% (Clinic 1, 2, 3) were from the antimicrobial class. These classes also represented the most commonly prescribed drug classes.

**Figure 2:**
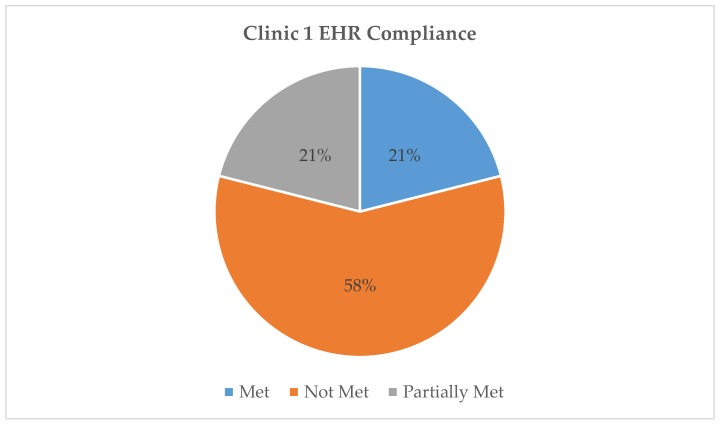
Clinic 1 electronic health record (EHR) compliance with AAP recommendations.

**Figure 3:**
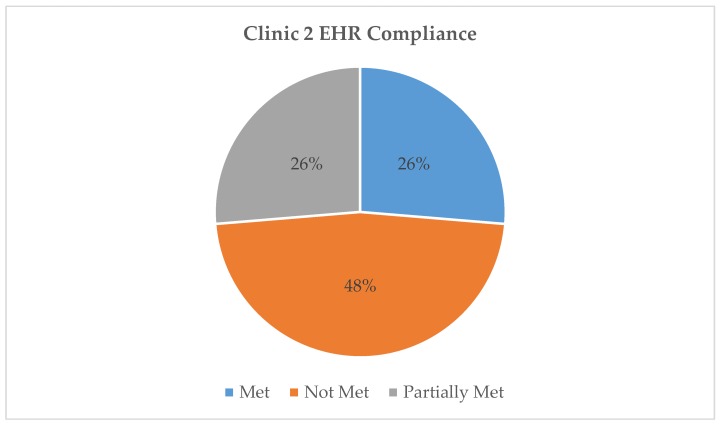
Clinic 2 EHR compliance with AAP recommendations.

**Figure 4:**
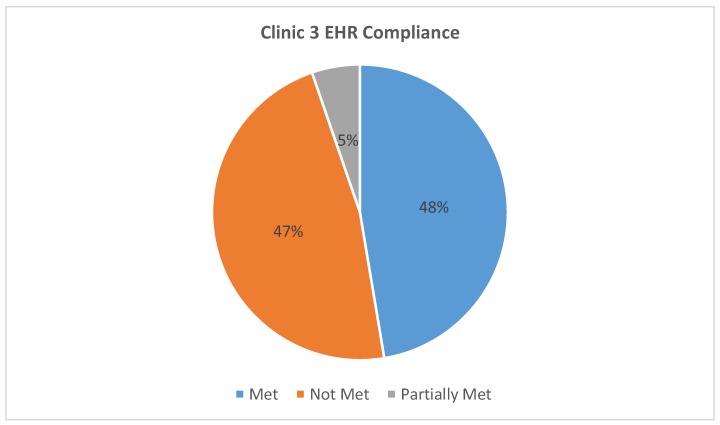
Clinic 3 EHR compliance with AAP recommendations.

All three EHR systems were assessed for compliance with the AAP requirements for safe and effective e-prescribing [11]. Table 1 represents a breakdown of each AAP requirement by clinic EHR and classifies them as “met”, “partially met”, or “not met”. For example, all three clinics met the requirement of “date of birth or age in units more specific than years” as this was present and functioning at the time of the testing. Clinic 1 partially met the “weight in kg” requirement because their EHR allowed the option of choosing between kilogram or pound as the weight unit. Lastly, no clinic met the cognitive support requirement of “adverse effect warnings specific to pediatric population” as these warnings were absent from all three EHRs. Our findings indicate that the EHR systems used by Clinics 1, 2, and 3 respectively fully met 21%, 26%, and 47%, of the AAP requirements for safe and effective e-prescribing. Figure 2, Figure 3 and Figure 4 show the total, numerical breakdown of AAP criteria per clinic.

**Figure 5:**
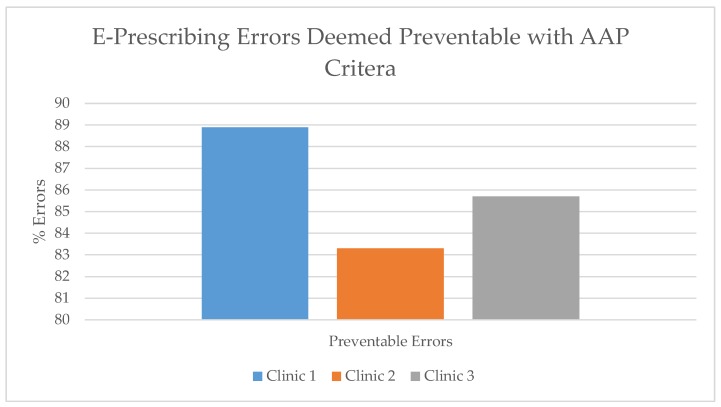
E-prescribing errors deemed preventable with AAP criteria.

Compliance with the AAP EHR recommendations could have prevented over 83% (range: 83.3–88.9%) of the medication errors at the clinics (Figure 5). A number of these errors were dosing errors (under dose or overdose) that could have been detected by the dose-range checking, cognitive support feature. An example of an error not avoided by AAP measures is albuterol 0.5% (2.5 mg/0.5 mL) with the instructions to give 2.5 mg (0.5 mL) in nebulizer every 3–4 h, as needed. The prescription is written for the concentrated solution that requires dilution with 3 mL normal saline. However, as written a caregiver or patient may simply put 0.5 mL in the nebulizer cup, causing an error and the inability to deliver the prescribed dosage.

## 4. Discussion

Children have unique needs compared to their adult counterparts when it comes to the delivery of safe and effective medical care. Their unique needs must be reflected in the EHR systems used to create and transmit e-prescriptions. This study highlights the problem of pediatric prescribing errors in the outpatient setting found when using an EHR and identifies errors that could have been avoided if an EHR had appropriate pediatric focused functionality. In 2013, AAP published their policy titled “Electronic prescribing in pediatrics: toward safer and more effective medication management” [11]. Using the AAP policy as a guide, this study evaluated real-life pediatric medication errors against recommendations identified within the AAP policy. This study indicates that over 83% of the prescribing errors found could have been avoided in an EHR platform aligned with AAP recommendations. Simply adding medication information including indication based dosing would have avoided dosing errors, as would have the addition of cognitive support including dose range checking.

To further assist in evaluating an EHR, AAP has created an EHR shopper’s guide for clinicians caring for children [14]. Providers should particularly prioritize EHR functionality that computes weight-based drug dosage and age/weight specific single dose range checking. Interestingly, within the priority to compute a weight-based drug dosage, it is possible to display the weight-based dosing strategy (when applicable) directly on the prescription, which would even further prevent dosing errors by allowing the filling pharmacist to accurately check the prescribed dosage.

Even an AAP compliant EHR would have failed to avoid up to 17% of errors identified in this study. A solution to some of these problems could involve EHR customization. The use of indication specific dosing and the inclusion of weight based dosing recommendations and calculations would resolve the majority of dosing errors. The use of a customized pediatric-clinic specific medication list within the EHR could additionally minimize errors with dosage form selection or dosage form specific dosing recommendations. For example, the albuterol dosage form error earlier identified would have been avoided if the prescriber had a customized pediatric list that included the commercially available albuterol 0.083% (2.5 mg/3 mL) vials. A custom medication list would also further promote proper medication selection.

On the other hand, some errors would not have been avoided even with full AAP compliance and custom lists. One error, for example, revealed an issue with the dosing calculator for certain ferrous sulfate products. In a 220 mg/5 mL oral solution formulation of ferrous sulfate, 44 mg/5 mL is elemental iron. Dosing should be based on this elemental (44 mg/5 mL) component, however the dose was calculated using 220 mg/5 mL leading to an 80% under dose. It is clear that EHR systems are further along in meeting AAP requirements related to patient information and medication information; while less equipped to meet cognitive support, pharmacy information or data transmission needs.

Limitations exist with this novel review of EHR shortcomings related to pediatric use. This study represents a retrospective, descriptive study that identifies and demonstrates a need for EHR systems to address pediatric needs. However, a larger, more vigorous assessment would be beneficial to help develop a more robust, statistically driven plan. In addition, each of the three clinics reviewed utilized different EHR systems so comparing error rates and types with different user groups of the same system may be enlightening and could reveal other issues including education differences with respect to system use. Finally, seasonal variation is not accounted for with the snapshot of prescriptions reviewed at the clinics.

With only a small percent of AAP criteria being met by each EHR used in the clinic setting today, there is much work to do in providing technology tools with the pediatric patient in mind. EHR systems should meet a minimum standard for pediatric support without requiring institution specific customization in order to provide appropriate safety checks and balances. Transmitting the patient’s weight to the pharmacy to allow dose checking; and providing mathematical conversion of fluid doses from mg/kg to mg then to milliliters per dose should be standard for all systems.

Lastly, more research is needed in the area of pediatric medication safety related to technology in the outpatient environment. A growing volume of data exists looking into EHR safety related to children in the hospital setting, but little data is available examining EHRs in the outpatient pediatric setting. Some error types are consistent between the inpatient and outpatient settings, such as improper dose. However, unique errors in the hospital setting such as wrong rate or monitoring errors are not applicable to our outpatient-focused study [15]. Of note, a study looking at three different pediatric health care institutions identified EHR usability issues (e.g., system feedback, visual display, data entry, and workflow support) as contributing causes of medication events [15]. This concept of usability would add value if further studied in EHRs used in the pediatric community setting.

## 5. Conclusions

This study demonstrates that EHR systems used by many pediatric clinic practices do not meet the standards set forth by the AAP. To ensure our most vulnerable population is better protected, it is imperative that medical technology tools adequately consider pediatric needs during development and be reflected in selected EHR systems.

## Figures and Tables

**Table 1:** American Academy of Pediatrics (AAP) requirements for safe and effective electronic prescribing [11].

Category	Pediatric Requirements	Clinic		
		1	2	3
Patient information	Date of birth or age in units more specific than years	M	M	M
	Weight in kg	P	M	M
	Height in cm	P	M	M
	Any history of intolerable adverse effects or allergy to meds	M	M	M
Medication information	Indication-based dosing	N	P	N
	Individual and daily dose alerts, using mg/kg per day or mg/m^2^ per day formula, unless inappropriate	N	P	M
	Weight-based dosing calculations	P	M	P
	All available formulations, including liquid formulations that may be specific brands	M	N	M
	Common formulations requiring extemporaneous compounding or combinations of active ingredients	N	N	N
Cognitive support	Dose range checking	P	P	M
	Automatic strength to volume conversion for liquid medications	N	P	M
	Adverse-effect warnings specific to pediatric populations	N	N	N
	Alternative therapies based on ameliorable adverse effects	N	N	N
	Tall-man lettering to reduce medication selection errors	N	N	N
	Medication-specific indications to reduce ordering of sound-alike drugs	N	N	N
Pharmacy information	Pharmacies that will create extemporaneous compounds	N	N	N
Data transmission	Use of messaging standards for data transmission to pharmacies that include the patient’s weight	N	N	N
	Use of messaging standards for data transmission to pharmacies that include notes pertaining to weight-based calculations	N	N	N
	Transmission of strength, concentration, and dose volume labeled in metric units for liquid medications	M	P	M

M = Met; P = Partially Met; N = Not Met.

**Table 2:** Types of medication prescribing errors [12].

Type	Examples
Incomplete/inadequate prescription	Directions missing Quantity sufficient (QS) with no day supply * Direction unclear Strength missing Quantity calculated in error Quantity missing Frequency missing
Dosing outside recommended range	Exceeds recommended dose Below recommended dose Frequency outside recommended Outside range for indication Duration outside recommended
Drug selection	Wrong dosage form Direction/dosage form mismatch Dosage form not available Not recommended for age Patient allergic to medication Wrong drug selected from list
Administration method	Technique not recommended Immeasurable dose

* To calculate QS the day supply is required to determine number of tablets to dispense. For example, one tablet twice a day for ten days. The QS would be 20 tablets.
